# Platform- and label-free detection of lead ions in environmental and laboratory samples using G-quadraplex probes by circular dichroism spectroscopy

**DOI:** 10.1038/s41598-020-77449-5

**Published:** 2020-11-24

**Authors:** Raeyeong Kim, Young-Sang Youn, Misook Kang, Eunjoo Kim

**Affiliations:** 1 Department of Chemistry, Yeungnam University, Gyeonsan, Gyeongbuk 38541 Republic of Korea; 2 Division of Electronic Information System Research, Daegu Gyeongbuk Institute of Science and Technology, Techno-jungangdaero 333, Daegu, 42988 Republic of Korea

**Keywords:** Chemical biology, Environmental sciences

## Abstract

Guanine-rich quadruplex (G-QD) are formed by conversion of nucleotides with specific sequences by stabilization of positively charged K^+^ or Na^+^. These G-QD structures differentially absorb two-directional (right- and left-handed) circularly polarized light, which can discriminate the parallel or anti-parallel structures of G-QDs. In this study, G-QDs stabilized by Pb^2+^ were analyzed by a circular dichroism (CD) spectroscopy to determine Pb^2+^ concentration in water samples. Thrombin aptamer (TBA), PS2.M, human telomeric DNA (HTG), AGRO 100, and telomeric related sequence (T2) were studied to verify their applicability as probes for platform- and label-free detection of Pb^2+^ in environmental as well as laboratory samples. Among these nucleotides, TBA and PS2.M exhibited higher binding constants for Pb^2+^, 1.20–2.04 × 10^6^/M at and 4.58 × 10^4^–1.09 × 10^5^/M at 100 micromolar and 100 mM K^+^ concentration, respectively. They also exhibited excellent selectivity for Pb^2+^ than for Al^3+^, Cu^2+^, Ni^2+^, Fe^3+^, Co^2+^, and Cr^2+^. When Pb^2+^ was spiked into an effluent sample from a wastewater treatment plant (WWTP), its existence was detected by CD spectroscopy following a simple addition of TBA or PS2.M. By the addition of TBA and PS2.M, the Pb^2+^ signals were observed in effluent samples over 0.5 micromolar (100 ppb) concentration. Furthermore, PS2.M caused a Pb^2+^-specific absorption band in the effluent sample without spiking of Pb^2+^, and could be induced to G-QD structure by the background Pb^2+^ concentration in the effluent, 0.159 micromolar concentration (3.30 ppb). Taken together, we propose that TBA and PS2.M are applicable as platform- and label-free detection probes for monitoring Pb^2+^ in environmental samples such as discharged effluent from local WWTPs, using CD spectroscopy.

## Introduction

Among toxic metals, lead is a well-known anthropogenic contaminant, which is discharged into the environment during the disposal of lead-containing consumer products, metal manufacturing processes, fossil fuel combustion, and sewage treatment and disposal processes^1–4^. Although lead contamination has been strictly regulated all over the world, the use of Pb-based products has increased owing to the world-wide development and application of solar cells, Pb–acid batteries, and radioisotope shields, etc^5–7^. It is notable that lead pollution does not only occur locally but also worldwide due to its diverse usages.

It has been reported that lead toxicity on human and animal species can cause various harmful effects including biochemical abnormalities, gastrointestinal diseases, impaired growth, cognition problems, and death^8–11^. For the support of the technology to reduce lead poisoning, rapid and simple detection of Pb^2+^ in laboratory and environmental samples is required to improve the existing time- and labor-consuming techniques such as inductively coupled plasma mass spectrometry (ICP) or atomic absorption spectroscopy.

**Figure 1:**
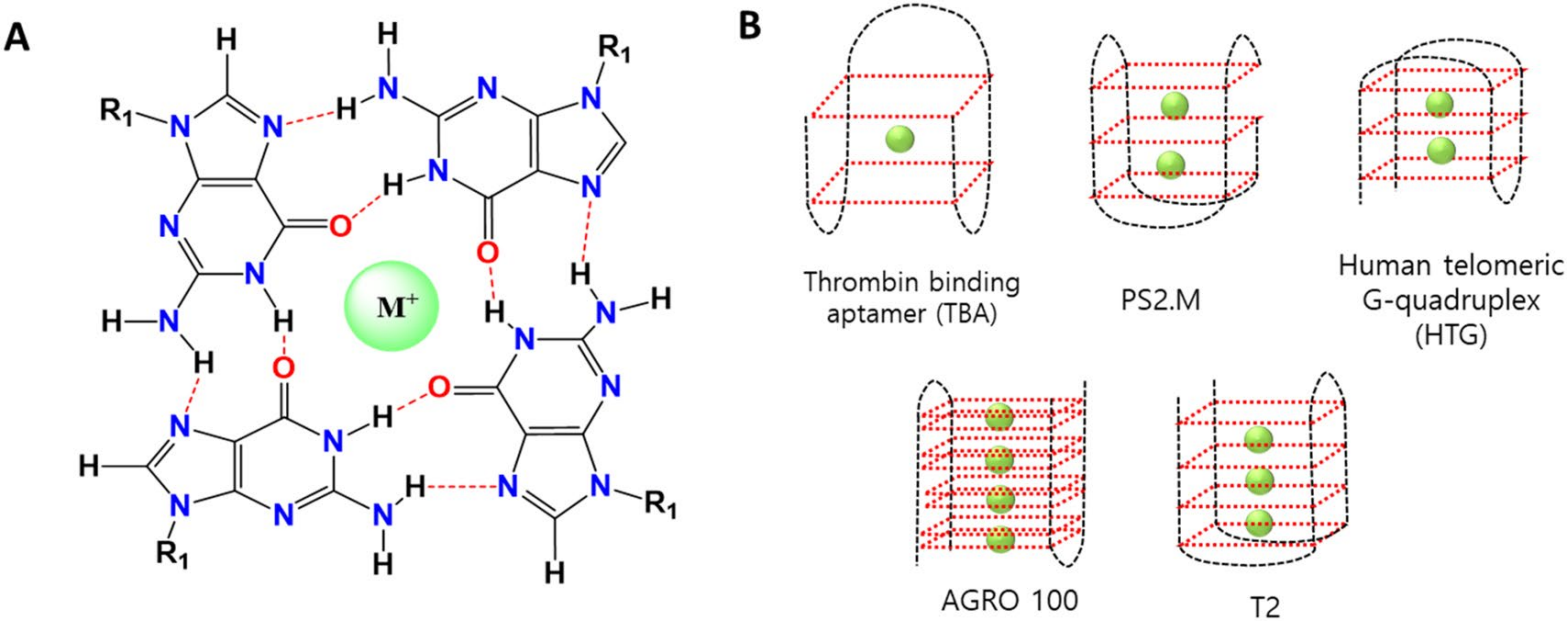
Guanine-rich quadruplex structures (G-QDs) investigated in this study. (**A**) Chemical structure of G-QDs involved with a metal ion. (**B**) Schematic design of G-QDs for five types of nucleotides. Green circles represent metal ions, such as K^+^, Na^+^, or Pb^2+^.

For the detection of Pb^2+^, we considered the use of self-reacting guanine-rich quadruplexes (G-QDs) as a simple probe that does not require any sensing platform and labeling reagents. G-QDs are a class of DNA secondary structures composed of four-stranded DNA structures^12^. For the last few decades, G-QDs have been reported for their biological relevance, including their participation in the protection of chromosomes by telomeres and in the control of gene expression^13^. For these biological processes of G-QDs, the unfolded DNA with guanine-rich sequences must be stabilized by the binding of cations (typically K^+^ or Na^+^) and switched to quadruplex secondary structures (Fig. 1A,B)^14^. The resulting G-QDs with the four-stranded secondary structure have high affinity and selectivity toward specific analytes such as nucleic acids, proteins, small molecules, and metal ions^15,16^.

Until now, various nucleotides that can be switched to G-QDs by K^+^ stabilization have been reported, such as thrombin aptamer (TBA), PS2.M, human telomeric DNA (HTG), AGRO 100, and telomeric related sequences (T2)^17–21^. In addition, it has been reported that Pb^2+^ can replace K^+^ during the switching of guanine-rich sequences to G-QDs. TBA, PS2.M, HTG, and T2 exhibited a conversion to G-QDs by Pb^2+^, which was measured by a circular dichroism (CD) spectroscopy^21–24^. As an application of these properties to sensor preparation, HTG induced by Pb^2+^ is utilized as a radon sensor with organic dye malachite green^25^. In addition, Pb^2+^ and hemin were co-stabilized in the AGRO 100 quadruplex structure, and Pb^2+^ could be detected by fluorescent, colorimetric, and electrochemical methods because of the optical and electrical properties of hemin^26^. Recently, fluorophore-labeled G-QDs have been reported to detect metal ions including Pb^2+^. Peng et al. used the T30695 sequence [d(GGGT)_4_] for Pb^2+^ detection^27^. They applied a fluorescent ratiometric method for Pb^2+^ detection by inserting Zn^2+^ to nucleotides, with a detection limit of 23.5 nM Pb^2+^. In addition, a perylene moiety was inserted at different phosphate positions of TBA without a significant effect on the G-QD structure, and their fluorescence anisotropy signals showed good linear relationship to the Pb^2+^ concentrations, with 24.5 nM detection limit^28^. The TAQ [d(TAG_3_T)_3_TAG_3_] sequence was also reported as a Ba^2+^ sensor with excellent tolerance to highly concentrated K^+^ by addition of a fluorophore, hypericin^29^.

However, the performance of these nucleotides for the detection of Pb^2+^ based on CD spectroscopy has not been successfully determined in environmental and laboratory samples as a Pb^2+^ sensor. The CD spectrum of G-QDs provides distinct binding geometry information and confirms the presence of different stacked bonds because of different orientations of guanine with respect to the glucoside bond^30^. For example, HTG typically shows a peak wavelength that is centered near 260 nm in CD spectrum exhibiting absorption difference (ΔA) caused by right- and left-handed circularly polarized light. However, if HTG switched to the G-QD structure by the K^+^ ion, it displays an absorption spectrum with a peak wavelength near 295 nm. CD spectroscopy can sensitively determine nucleic acid secondary structures and is particularly well-suited for monitoring structural changes resulting from folding-unfolding reactions caused by binding of specific ions to each nucleotide.

Herein, we collectively analyzed and compared the performance of the five types of G-QDs, TBA, PS2.M, AGRO 100, HTG, and T2, which have different DNA sequences, as an easy and rapid Pb^2+^ detection sensor using CD spectroscopy. The detection of Pb^2+^ was performed based on the degree of absorption caused by right- and left-handed circularly polarized light (ΔA) for Pb^2+^-incorporated G-QDs. The specific wavelength of the absorption peak was determined according to the structure of the G-QDs complexed by Pb^2+^. The sensing of Pb^2+^ complexed with G-QDs by CD spectroscopy does not require any labeling materials such as fluorescence dyes or other optical probes. It has been reported that fluorescence dye-labelled G-QDs were typically used for the application of G-QDs as sensors^31–33^.

In this study, the sensing performance of G-QDs was compared in terms of the absorption band, ΔA intensity at peak wavelength, association constant (K_A_), and cross-reactivity with other metal ions. Moreover, Pb^2+^ was spiked into an effluent of a wastewater treatment plant (WWTP) to demonstrate the applicability of G-QDs for the measurement of Pb^2+^ concentrations in environmental as well as in laboratory samples. Because environmental samples are usually containing K^+^, the sensing capacity of G-QDs for Pb^2+^ was investigated in the presence of K^+^.

## Materials and methods

### Materials

The nucleotides listed in Table 1 were purchased from Cosmogenetech (Seoul, Korea). The concentration of oligonucleotides was measured by a Cary 100 UV spectropolarimeter (Australia) using the following extinction coefficients: TBA for ε_260nm_ = 147,300 M^−1^ cm^−1^^34^, PS2.M for ε_260nm_ = 184,300 M^−1^ cm^−1^^24^, HTG for ε_260nm_ = 228,500 M^−1^ cm^−1^^35^, AGRO100 for ε_260nm_ = 250,800 M^−1^ cm^−1^^36^, telomeric related sequence (T2) for ε_257nm_ = 213,400 M^−1^ cm^−1^^37^. Before conducting the experiments, all oligonucleotides in Tris-buffer were denatured at 90 °C for 10 min and cooled at room temperature. KNO_3,_ Al(NO_3_)_3_∙9H_2_O, Cu(NO_3_)_2_∙3H_2_O, Ni(NO_3_)_3_∙6H_2_O, Fe(NO_3_)_3_∙9H_2_O, Co(NO_3_)_2_∙6H_2_O, Cr(NO_3_)_2_∙9H_2_O, and Pb(NO_3_)_2_ were purchased from Sigma-Aldrich (St. Louis, MO, USA).

**Table 1:** Types of guanine-rich quadruplex structures (G-QDs) investigated in this study.

DNA Species	Sequence	Nucleotide number
Thrombin aptamer (TBA)	d(G_2_T_2_G_2_TGTG_2_T_2_G_2_)	15
PS2.M	d[GTG_3_TAG_3_CG_3_TTG_2_]	18
Human telomeric DNA (HTG)	d[AG_3_(T_2_AG_3_)_3_]	22
AGRO 100	d[(GGT)_4_TGT(GGT)_3_GG]	26
Telomeric related sequence (T2)	d(G_4_T_2_G_4_T_2_G_4_T_2_G_4_)	22

### CD spectroscopy for G-QD formation

The oligonucleotides were dissolved by 3 μM in 20 mM Tris–HCl (pH 7.0) without any Na^+^ and K^+^. The CD spectra were obtained from 220 to 400 nm by using a J-715 spectropolarimeter with a 1 × 1 cm quartz cuvette. (Tokyo, Japan); data interval: 1 nm; band width: 2 nm; scan speed: 50 nm/min; response time: 2 s. The base line was collected using a control sample without nucleotides and subtracted from each spectrum for the nucleotide samples.

K^+^ and Pb^2+^ were dissolved in deionized water (DW), and the final concentration was met by adding 200 μL of cation solution to 1.8 mL nucleotide solution.

### Determination of binding constants for Pb^2+^

To quantitatively determine the binding affinity, the binding constant of Pb^2+^ for G-QDs was determined using the Langmuir adsorption model^38^. The binding affinity was calculated by measuring the signal intensity (ΔA) in terms of milli-degree (mdeg) by CD spectroscopy, which was used as the numeric value of the response at equilibrium. The correlation of the Pb^2+^ concentration in this binding system and the response value at equilibrium can be expressed by the following equation.

1 $$\frac{C}{q} = \frac{C}{{q_{m} }} + \frac{1}{{q_{m} \cdot {\text{K}}_{A} }},$$

where q is the CD signal intensity (mdeg) at the peak wavelength of each G-QDs, and C is the Pb^2+^ concentration. K_A_ is the apparent binding constant, and q_m_ is the CD signal intensity when C is infinity. The binding constant was determined using data from three repetitive experiments.

### Measurement of selectivity of Pb^2+^ over other metal ions

The TBA and PS2.M nucleotides were dissolved by 3 μM in 20 mM Tris–HCl (pH 7.0) to measure the cross reactivity of other metal ions to TBA and PS2.M. Metal ions such as Al^3+^, Cu^2+^, Ni^2+^, Fe^3+^, Co^2+^, Cr^2+^, and Sr^2+^ were dissolved in DW at various concentrations, and the solution at each concentration was added by 200 μL to 1.8 mL of nucleotide solution. To determine the effect of multiple cations, metal ions used above was collectively dissolved in DW to meet 3 μM respectively, and 200 μL of the multiple cation solution was added to make in total 2 mL mixture with nucleotide solution. The measurement of CD spectroscopy was performed as described above.

### Detection of Pb^2+^ dissolved in effluent from a municipal wastewater treatment facility

Three effluent samples (Sample 1–3) of the WWTP was obtained from Korea Water Cluster (Daegu, Korea). Before detection, each effluent sample was filtered through a 0.2-μm membrane. TBA and PS2.M was dissolved to 3 μM concentration in 20 mM Tris–HCl (pH 7.0). Pb^2+^-spiked effluent was prepared by dissolving Pb^2+^ from 0 to 120 μM concentration in an effluent sample (Sample 1). Each Pb^2+^ solution was added by 200 μL to 1.8 mL of the nucleotide solution. The recovery of the signal intensity by spiked Pb^2+^ in the Sample 1 was calculated by division of the intensity at 314 nm by the intensity of the standard solution prepared by dissolving Pb^2+^ in DW. To detect the existence of Pb^2+^ in three effluent samples without spiking of Pb^2+^, CD intensity was measured by adding 200 μL of each effluent to 3 μM nucleotide solution dissolved in 20 mM Tris–HCl (pH 7.0).

The concentration of metal ions in the effluent from the WWTP was analyzed through inductively coupled plasma optical emission spectrometry (ICP-OES; iCAP7400, Thermo Scientific, Waltham, MA, USA).

## Results and discussion

### Characteristics of CD spectrum upon binding of Pb^2+^ to G-QDs

**Figure 2:**
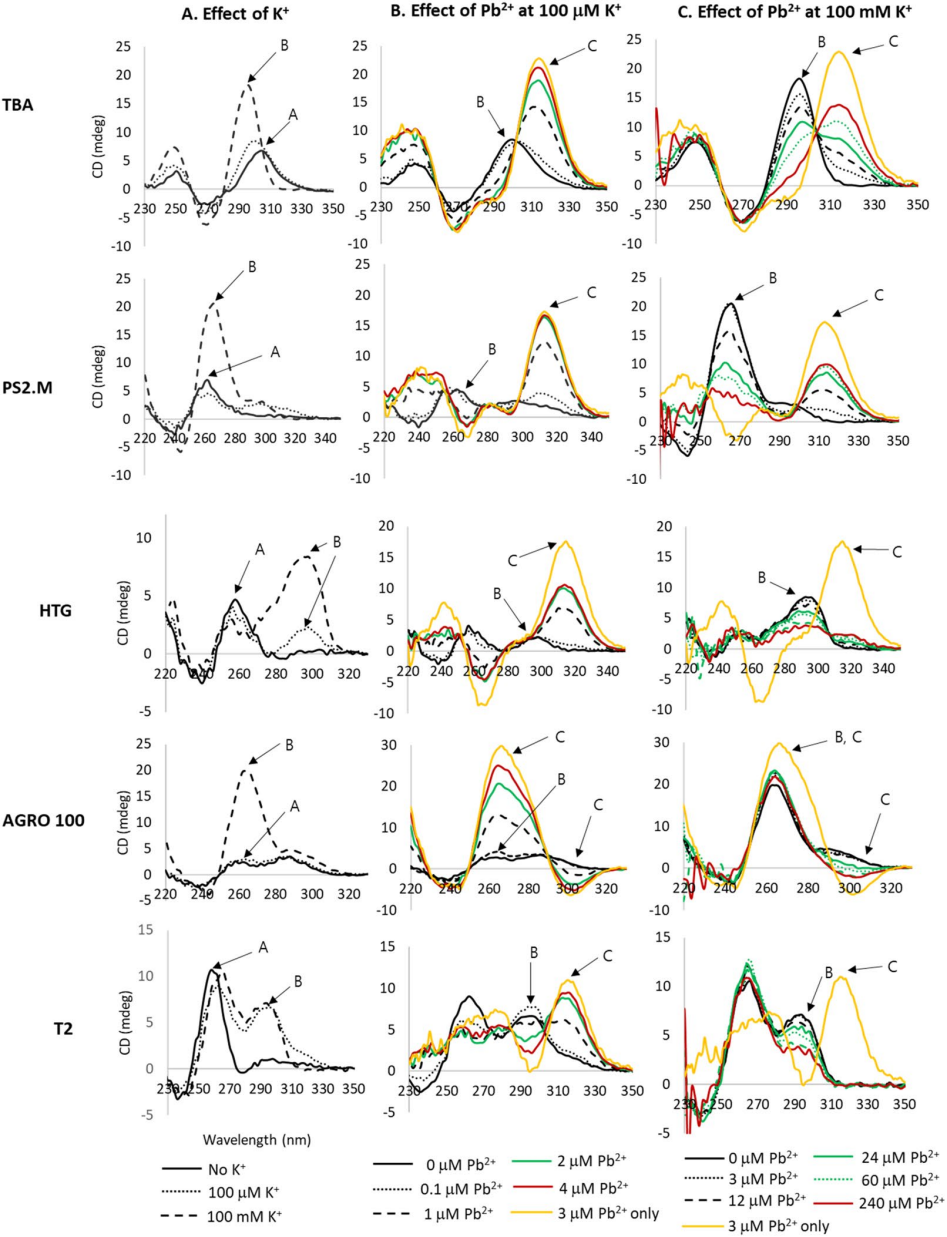
Circular dichroism (CD) spectra G-QDs by addition of K^+^ and Pb^2+^. (**A**) Structural changes observed in five types of G-QDs by addition of K^+^. (**B**,**C**) Structural changes observed by incorporation of Pb^2+^. (**B**) Effect of Pb^2+^ at 100 μM K^+^; (**c**) effect of Pb^2+^ at 100 mM K^+^. Yellow line is the spectra induced by Pb^2+^ without K^+^.

CD spectroscopy was employed to examine the K^+^ and Pb^2+^-induced G-QD structures (Fig. 2). Peak [A] indicates the absorption peak of each G-QD without K^+^, whereas peak [B] indicates the absorption peak induced by K^+^. The wavelengths of the peaks [B] for TBA and PS2.M were slightly shifted from peak [A], to − 8 and + 4 nm, respectively. For peak [B], a dramatic increase in the absorption intensity was observed at 100 mM K^+^. However, a slight increase for TBA and no shift for PS2.M were observed at 100 μM K^+^ concentration, and new absorption bands near 298 and 294 nm were observed for HTG and T2, respectively, at both 100 μM and 100 mM K^+^ concentration (Fig. 2A). The absorption band of AGRO 100 near 264 nm was not shifted by the addition of K^+^; however, the peak intensity at 264 nm was dramatically increased at 100 mM K^+^ concentration. This result indicated that the switched form of AGRO 100 induced by K^+^ ions had the same peak wavelength as the denatured AGRO 100, and the CD signal intensity was enhanced by complexation with K^+^ at 100 mM concentration.

The shift of the peak wavelength ([B]-[A]) for each unfolded nucleotide ([A]) and new peaks by K^+^ complexation ([B]) are summarized in Table 2. The absorption peaks of these G-QDs by K^+^ complexation were similar to those reported previously for each G-QD^19–23^.

**Table 2:** CD spectral characteristics for each G-QD owing to addition of K^+^ and Pb^2+^.

Type	Free nucleotide	G-QD by K^+^		G-QD by Pb^2+^		
	Peak (nm) [A]	Peak (nm) [B]	Shift [B]–[A]	Peak (nm) [C]	Shift [C]–[A]	Shift [C]–[B]
TBA	304	296	− 8	314	+ 10	+ 18
PS2.M	262	266	+ 4	314	+ 50	+ 46
HTG	258	298	+ 40	314	+ 56	+ 16
AGRO100	264	264	0	264/304^a^	0/+ 40	0/+ 40
T2	258	294	+ 36	314	+ 58	+ 22

^a^For AGRO 100, Pb^2+^ ions induced a negative increase in the peak height at 304 nm according to the Pb^2+^ concentration, which is a unique wavelength for Pb^2+^. A positive increase in peak height was observed at 264 nm, which was identical to the response of K^+^ and Pb^2+^. In this study, negatively increased peaks were considered as an original response to Pb^2+^

Peak [C] indicated the absorption peak upon addition of Pb^2+^ (Fig. 2B,C). The Pb^2+^-originated absorption wavelength ([C]) was shifted from the K^+^-induced peak ([B]) for TBA, PS2.M, HTG, AGRO 100, and T2 by 18, 46, 16, 0/40, and 22 nm, respectively ([C]-[B] in Table 2). The peak wavelength of Pb^2+^-induced AGRO 100 was observed at two wavelengths—264 and 304 nm. The peak intensity at 264 nm was positively related to the concentration of Pb^2+^, but it was the same as that for the peak induced by K^+^ ions. The absorbance at 304 nm gave a unique signal for Pb^2+^ incorporation into AGRO 100, and the signal intensity decreased with increasing Pb^2+^ concentration. In this study, we investigated the capability of a G-QD sensor for the specific binding of Pb^2+^; thus, the intensity of the peak at 304 nm was considered to be only a Pb^2+^-induced response. In Fig. 2B,C, yellow lines indicate the spectra induced by Pb^2+^ without the addition of K^+^. The transition of G-QDs were induced by Pb^2+^, therefore, the intensity of peaks induced by Pb^2+^ only was similar or higher to those of Pb^2+^ with 100 μM K^+^.

All G-QDs studied herein formed Pb^2+^-induced structures, and the signals were clearly discriminated from the signals by K^+^. Therefore, the CD signal could facilitate the detection of Pb^2+^ in water samples, which contained K^+^ ions near 100 μM. When K^+^ concentration was increased to 100 mM, the switched structures of TBA, PS2.M, HTG and AGRO 100 by Pb^2+^ were also observed distinctly from those of K^+^, but the CD signal intensity was not high as observed for that at 100 μM K^+^ concentration (Fig. 2C). Moreover, the T2 absorption band by Pb^2+^ disappeared at 100 mM K^+^ concentration. It was attributed to the Pb^2+^ binding affinity to T2 that was not sufficient to stabilize the G-QD structure at high K^+^ concentration. The stability of Pb^2+^ binding, instead of K^+^, depended on the structural differences of G-QDs, which was originated from the nucleotide sequences.

Taken together, there are specific absorption bands induced by the incorporation of Pb^2+^ ([C]), which are completely different from those of K^+^-induced G-QDs ([B]) as well as unfolded G-QDs ([A]). The clear absorption band of G-QDs specifically induced by Pb^2+^ implied that a unique conformation of G-QDs was constructed by the addition of Pb^2+^. The results indicated that the intensity of the CD signal at the absorption band could be used for the signal of the Pb^2+^ sensor, reflecting its concentration in the samples.

### Determination of maximum binding and association constants of Pb^2+^ to G-QDs

**Figure 3:**
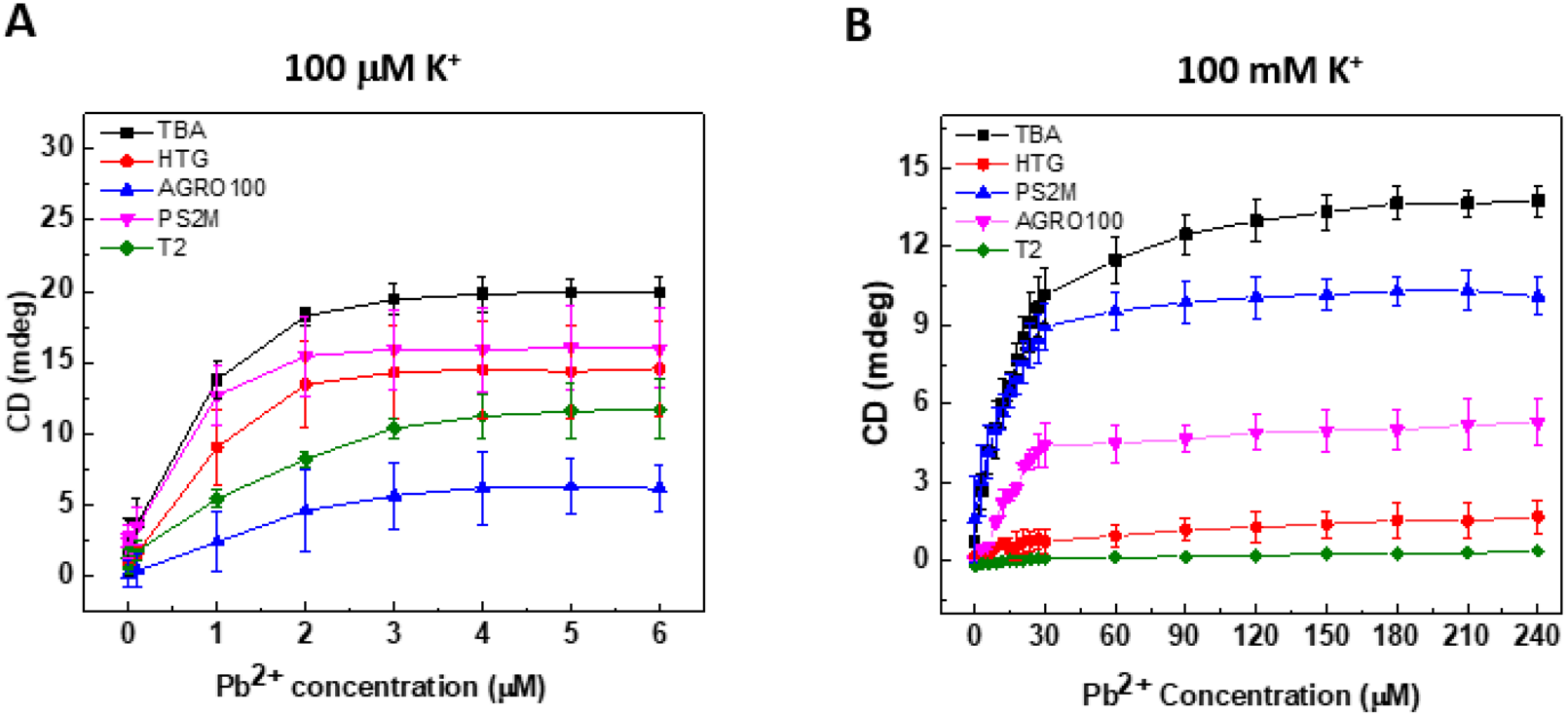
Concentration-dependent CD spectra. (**A**) Pb^2+^-dependent CD response at 100 μM K^+^ concentration. (**B**) Pb^2+^-dependent CD response at 100 mM K^+^ concentration.

Figure 3 shows the relationship between the Pb^2+^ concentration and the intensity of the CD signal at the peak wavelength [C] in Fig. 2. TBA and PS2.M exhibited higher CD signal intensity at both 100 μM and 100 mM K^+^. At 100 μM K^+^ concentration, the signal increased from 100 fM Pb^2+^, and the binding capacity was saturated from 3 μM (Fig. 3A). However, Pb^2+^ was detected at 3 μM concentration and saturated from 60 μM Pb^2+^ at 100 mM K^+^ concentration (Fig. 3B). TBA showed a delayed saturation compared to other nucleotides. This result indicated the importance of K^+^ concentration in the Pb^2+^ sample solution, because Pb^2+^ should compete with K^+^ at high K^+^ concentration. Several studies reported that replacement of K^+^ by Pb^2+^ in a quadruplex was observed^22,24^, which supported that K^+^ concentration could affect the binding affinity of Pb^2+^ in sensing process.

**Figure 4:**
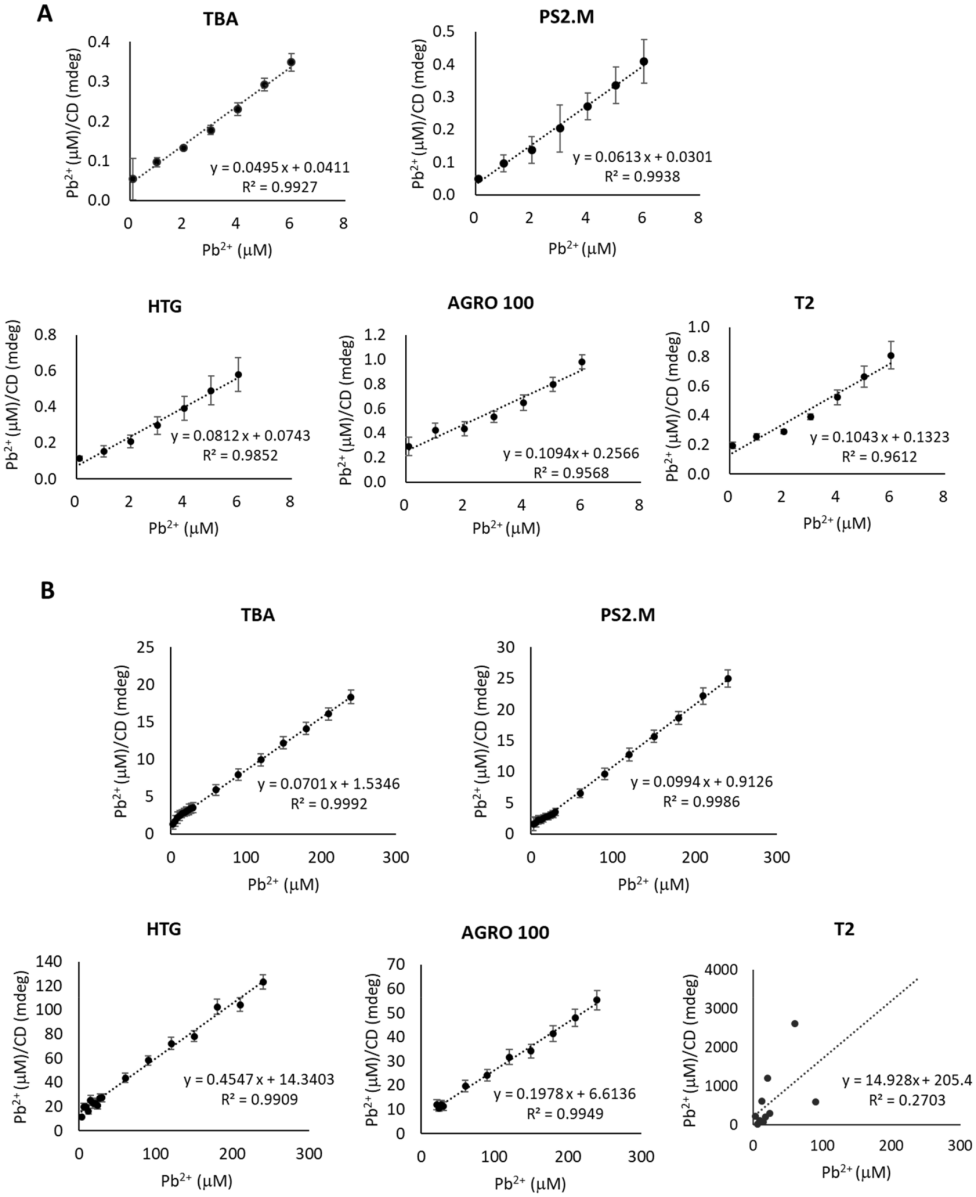
Plot of Langmuir isotherm equation for the calculation of association constant (K_A_) of G-QDs for Pb^2+^. The plot was prepared by c vs c/q. (**A**) Association relationship for each G-QD at 100 μM K^+^ concentration. (**B**) Association relationship for each G-QD at 100 mM K^+^ concentration. c, Pb concentration; q, CD absorption response.

To quantitatively determine the binding affinity, we calculated the binding constant of Pb^2+^ for G-QDs using the Langmuir adsorption model^38^. The plot according to Eq. (1) generated a straight line and the ratio of slope to intercept was K_A_, and the inverse slope was q_m_ (Fig. 4). In Fig. 4A, the linearity of the plots (R^2^) for the five G-QDs was 0.961–0.994 at 100 μM K^+^ concentration. However, the plots for AGRO 100 and T2 at 100 mM K^+^ concentration were not available to determine the binding constant, due to the negative intercept (AGRO 100) and poor C vs. q relationship (T2) (Fig. 4B).

The value of K_A_ for Pb^2+^ binding to G-QDs was determined as shown in Table 3. The binding constant of G-QDs at 100 μM K^+^ concentration was several orders of magnitude (10–100 fold) higher than that measured at 100 mM K^+^ concentration. The binding constants of G-QDs at 100 μM K^+^ concentration were also higher than those of other Pb^2+^ sensors, such as the surface plasmon resonance biosensor (2.4 × 10^2^–7 × 10^5^/M)^39^. In a recent study, a fluorophore was synthesized and the Pb^2+^ concentration was measured by fluorescence spectroscopy, and the binding constant was 5 × 10^5^/M^40^, comparable or lower than that calculated in this study. Taken together, the PS2.M structure had the highest association constant for Pb^2+^ (1.09 × 10^5^/M) even when there was a high concentration of competitive K^+^ up to 100 mM. TBA showed the highest q_m_ among the five G-QDs at both 100 μM and 100 mM K^+^ concentrations, which indicated that TBA could have the largest binding capacity for Pb^2+^ by forming a stable G-QD structure among the five G-QDs used in this study. Thus, TBA and PS2.M are considered as more suitable sensing materials for Pb^2+^ detection as an additional labeling probe and a platform are not required to immobilize them.

**Table 3:** Association constants of G-QDs for the binding of Pb^2+^.

G-QD	100 μM K^+^		100 mM K^+^	
	R_max_ (mdeg)	K_A_ (/M)	R_max_ (mdeg)	K_A_ (/M)
TBA	20.20	1.20 × 10^6^	14.27	4.58 × 10^4^
HTG	12.31	1.09 × 10^6^	2.20	3.18 × 10^4^
AGRO100	9.14	4.26 × 10^5^	–	–
T2	9.62	7.88 × 10^5^	–	–
PS2.M	16.31	2.04 × 10^6^	10.06	1.09 × 10^5^

### Selectivity of Pb^2+^ over other metal ions

**Figure 5:**
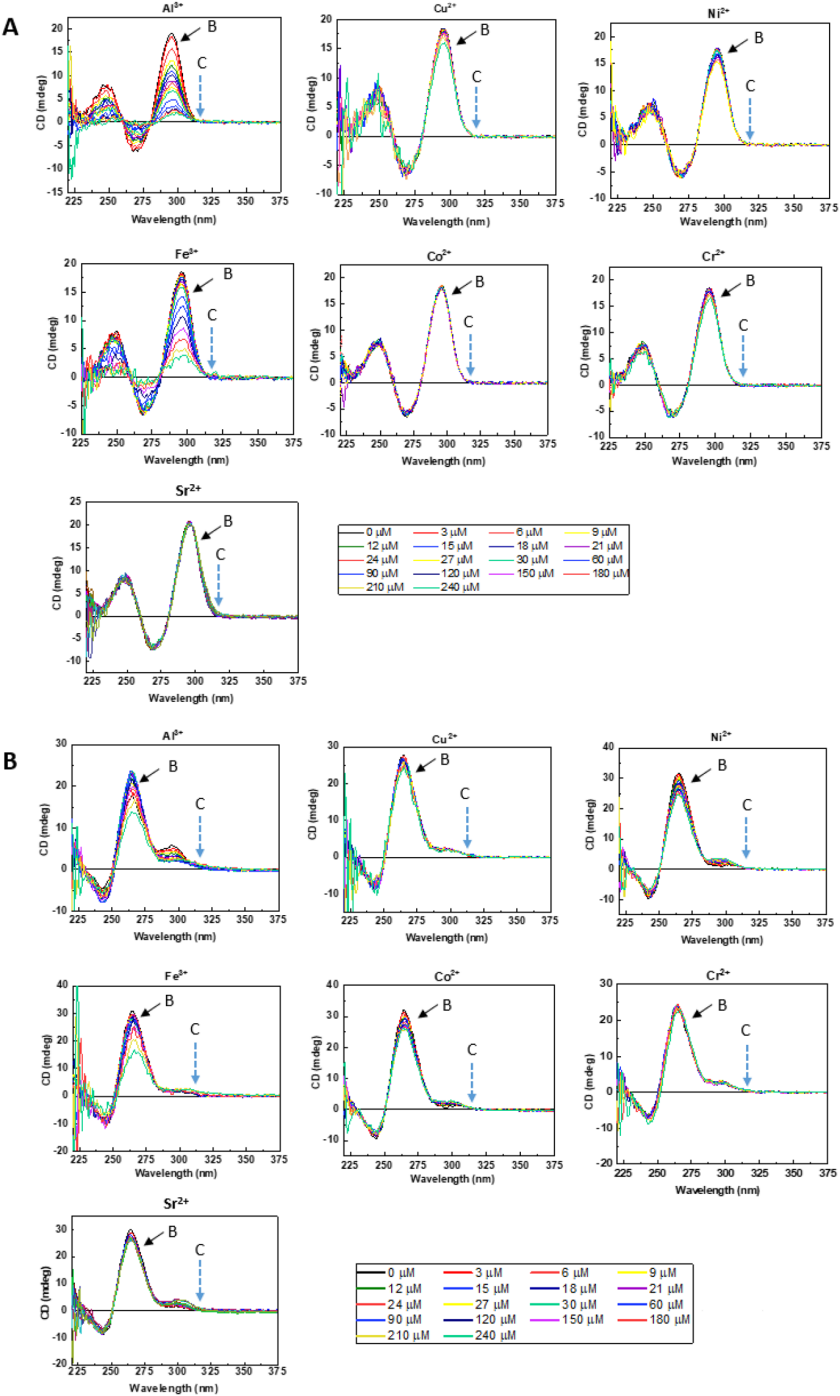
Cross-reactivity of G-QDs to metal ions. (**A**) Cross-reactivity of metal ions to TBA. (**B**) Cross-reactivity of metal ions to PS2.M. Black arrows indicate the K^+^-induced peak. Blue-dot arrows indicate the wavelength of Pb^2+^-induced G-QDs (314 nm for both G-QDs).

The specificity of Pb^2+^ for TBA and PS2.M, which were selected as the most efficient sensing materials among the five G-QDs investigated in this study, was investigated with other metal ions. As demonstrated in Fig. 5A (TBA) and 5B (PS2.M), neither G-QDs responded to other metal ions such as Al^3+^, Cu^2+^, Ni^2+^, Fe^3+^, Co^2+^, Cr^2+^, and Sr^2+^. The result exhibited a highly selective structural conversion by Pb^2+^ in contrast to the above-mentioned metal ions. The peak [B] indicates the peak induced by K^+^ ([B] in Fig. 2), and the peak [C] indicates the wavelength of absorption by Pb^2+^-induced G-QDs ([C] in Fig. 2).

For TBA, the specific wavelength of the CD peak caused by K^+^ complexation was 294 nm, and the peak height at 294 nm was decreased with increasing concentrations of Al^3+^ and Fe^3+^ (Fig. 5A). This was attributed to the deconstruction of the K^+^-specific structure owing to the addition of Al^3+^ and Fe^3+^ ions. However, the CD signal intensity at Pb^2+^-specific wavelength (314 nm) was not observed, which indicated that Al^3+^ and Fe^3+^ could not induce the structural conversion, as done by Pb^2+^, even if they could affect the binding of K^+^. All other ions such as Cu^2+^, Ni^2+^, Co^2+^, Cr^2+^, and Sr^2+^ could not affect the K^+^-specific binding of TBA and did not induce structural conversion as Pb^2+^ did.

**Figure 6:**
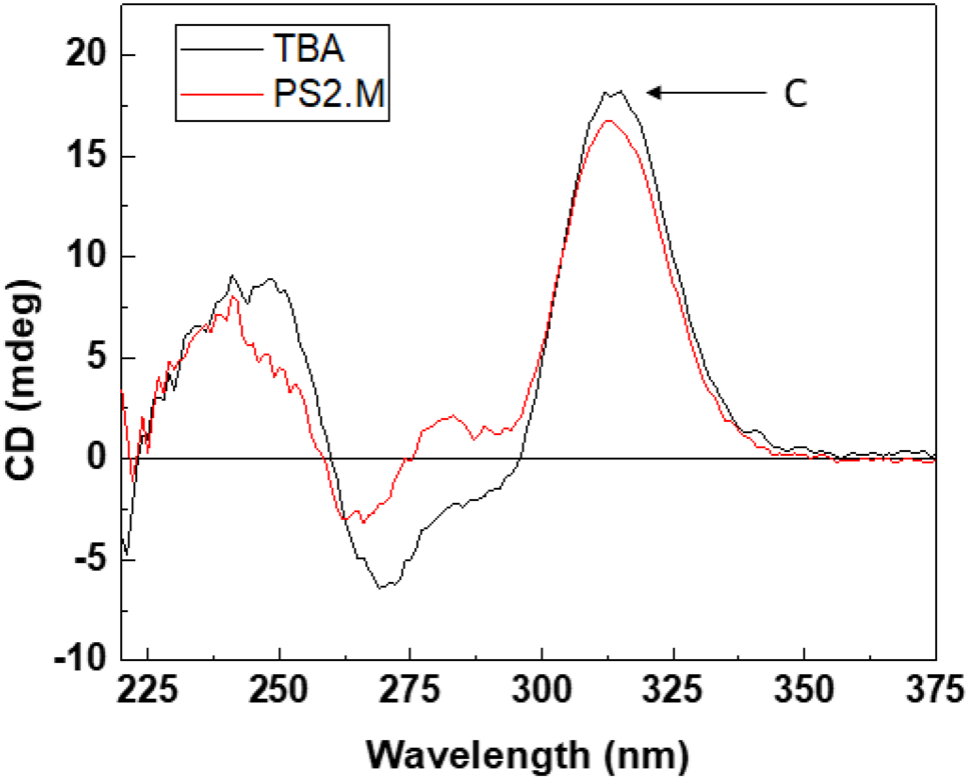
CD spectra of TBA and PS2.M by Pb^2+^ in the presence of multiple ions, Al^3+^, Cu^2+^, Ni^2+^, Fe^3+^, Co^2+^, Cr^2+^ and Sr^2+^. Each ion concentration including Pb^2+^ is 3 μM, without K^+^. Peak C indicated the 314 nm peaks of two G-QDs induced by Pb^2+^.

For PS2.M, the main peak at 266 nm originated from the structural conversion by K^+^ (Fig. 5B). The addition of Al^3+^, Ni^2+^, and Fe^3+^ to PS2.M decreased the peak height with increasing ion concentration. Although slight destruction of K^+^-induced G-QDs was observed by Al^3+^, Ni^2+^, and Fe^3+^, a Pb^2+^-specific peak that should be observed at 314 nm did not appear. This result indicated that PS2.M also had high specificity to Pb^2+^. Figure 6 shows the spectra of TBA and PS2.M induced by Pb^2+^ under the co-existence of seven ions. The result indicates that multiple ions did not interfere the transition of G-QDs by Pb^2+^.

**Figure 7:**
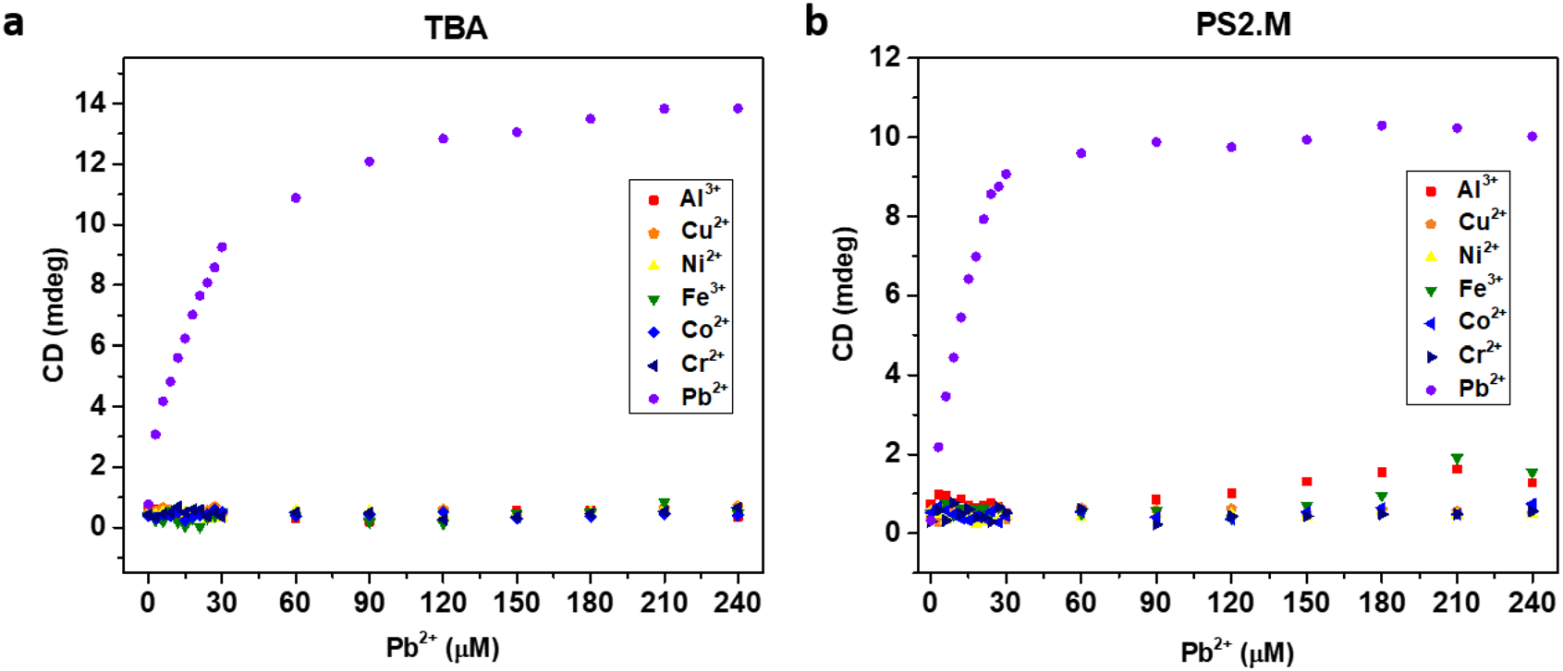
Cross-reactivity of TBA and PS2.M to other metal ions at 314 nm. (**a**) Cross-reactivity of metal ions to TBA. (**b**) Cross-reactivity of metal ions to PS2.M.

Figure 7 shows the intensity of the CD signal according to the metal ion concentration, at which TBA (Fig. 7A) and PS2.M (Fig. 7B) were switched to G-QD structures (314 nm) by Pb^2+^. As shown in Fig. 7, the structural transition of TBA and PS2.M induced by Pb^2+^ was clearly observed, and seven metal ions—Al^3+^, Cu^2+^, Ni^2+^, Fe^3+^, Co^2+^, Cr^2+^, and Sr^2+^—could not induce a structural transition. The selective transition at the Pb^2+^-specific wavelength could be a critical property of TBA and PS2.M as specific detection sensors of Pb^2+^.

### Analysis of Pb^2+^ spiked into wastewater samples

In order to test whether the proposed strategy for Pb^2+^ detection could be applied to environmental samples, the concentration of Pb^2+^ was analyzed in effluent samples of a WWTP located in Daegu, Korea. For our reference, the concentrations of metal ions in the sample were measured by ICP analysis including Pb^2+^ and K^+^ (Table 4). As shown in Table 4, the concentration of K^+^ in wastewater was 616 μM (Sample 1), 484 (Sample 2), and 214 μM (Sample 3), which could facilitate the slight transition of TBA to K^+^-dependent structures, as shown in Fig. 2A. For PS2.M, it was not expected that the transition of the K^+^-specific structure was occurred in the effluent samples, because the transition by 100 μM K^+^ concentration was not observed in Fig. 2A.

**Table 4:** Metal concentrations determined by ICP-OES analysis in the wastewater samples.

Metal	Sample 1		Sample 2		Sample 3	
	μM	ppb	μM	ppb	μM	ppb
K	616	24,100	482	18,8000	214	8402
Pb	0.0159	3.30	0.00676	1.40	N.D	N.D
Fe	1.55	86.3	1.98	110	0.00538	0.300
Al	12.7	342	5.04	136	N.D	N.D
Cu	N.D	N.D	0.279	17.7	N.D	N.D
Ni	0.0528	3.10	1.30	76.6	0.0528	N.D
Co	N.D	N.D	N.D	N.D	N.D	N.D
Cr	N.D	N.D	N.D	N.D	N.D	N.D

**Figure 8:**
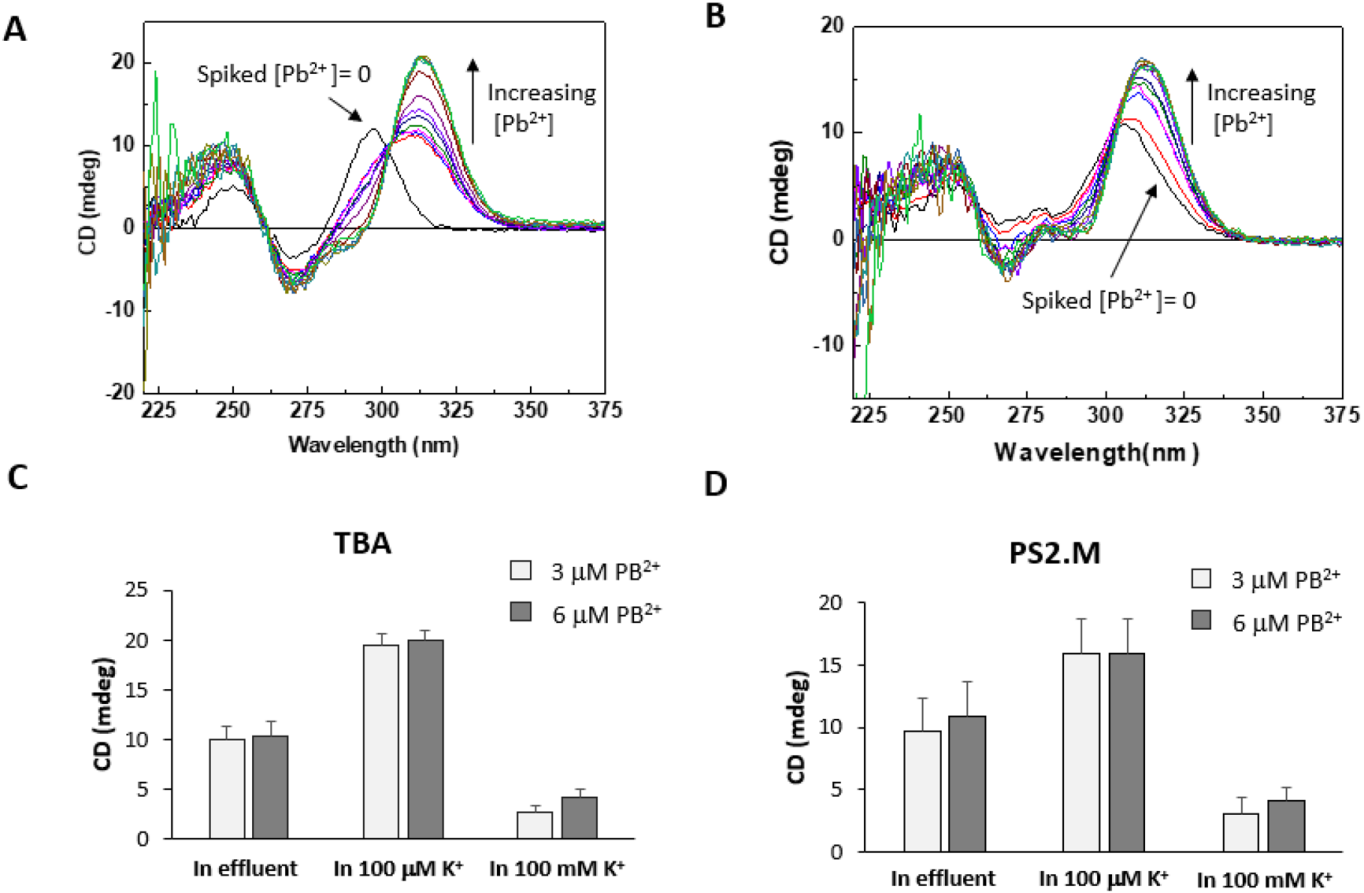
CD spectra of Pb^2+^-spiked effluent samples discharged from a local wastewater treatment plant (WWTP). Pb^2+^ detection using TBA (**A**) and PS2.M (**B**). Comparison of CD spectra of TBA (**C**) and PS2.M (**D**) added by Pb^2+^-spiked effluent samples and Pb^2+^in 100 μM and 100 mM K^+^ solution.

Pb^2+^ in the Sample 1 and Sample 2 was as low as 0.0159 μM (3.30 ppb) and 0.00676 μM (1.40 ppb), and in Sample 3, Pb^2+^ was not detected. To investigate the peak transition and recovery of CD signals in the effluent samples, Pb^2+^ was spiked into the Sample 1 from 0 to 120 μM. When Pb^2+^-spiked Sample 1 was added to the TBA solution, the transition of the TBA structure by Pb^2+^ was occurred at 314 nm, and the CD intensity was increased according to the Pb^2+^ concentration from 3 μM (Fig. 8A). The peak wavelength at 0 μM Pb^2+^ was 296 nm, which was identical to peak [B], the K^+^-induced transition. Thus, TBA could not detect the background Pb^2+^ concentration in the effluent sample (0.0159 μM). However, if the Pb^2+^ existed over 3 μM, it would be possible to detect Pb^2+^ dissolved in complex environmental samples.

On the other hand, the switching of PS2.M by K^+^ did not occur in the effluent Sample 1, as expected by the result in Fig. 2 (Fig. 8B). However, the CD spectrum shifted toward a Pb^2+^-specific transition wavelength (314 nm) due to the background Pb^2+^ in the effluent was observed (spiked [Pb^2+^] = 0). In addition, the CD signal intensity at 314 nm displayed a positive relationship with the spiked Pb^2+^ concentration.

The CD signal at 314 nm in the effluent Sample 1 was compared to the CD intensity for Pb^2+^-induced G-QDs with 100 μM and 100 mM K^+^(Fig. 8C,D), respectively. For both TBA and PS2.M, the CD intensity for Pb^2+^ in the effluent sample was decreased compared to that of Pb^2+^ in 100 μM K^+^, and increased to that of Pb^2+^ in 100 mM K^+^. The CD intensity at 314 nm by TBA and PS2.M in the effluent sample was 0.52- and 0.78-fold decreased than that of 100 μM K^+^ solution, respectively. However, they were increased to 3.1 and 2.9 folds to that of 100 mM K^+^ solution for TBA and PS2.M, respectively. The high recovery ratio of Pb^2+^ signal in effluent sample when comparing in 100 mM K^+^ solution was attributed to the low concentration of K^+^ in effluent sample, because K^+^ competes to Pb^2+^ as shown in Fig. 2B,C. However, the ratio of CD intensity of Pb^2+^-induced TBA in the effluent sample to 100 μM K^+^ solution (0.52) was more reduced than that of PS2.M (0.78). These results suggest that PS2.M could determine the Pb^2+^ concentration more efficiently, even though the samples were composed of complex materials released to municipal wastewater. In Supplementary Fig. S1, the Pb^2+^-induced spectra of TBA and PS2.M in 616 μM K^+^ was compared to those of 100 μM K^+^. The peak intensity and the shape of spectrum was almost the same between them.

**Figure 9:**
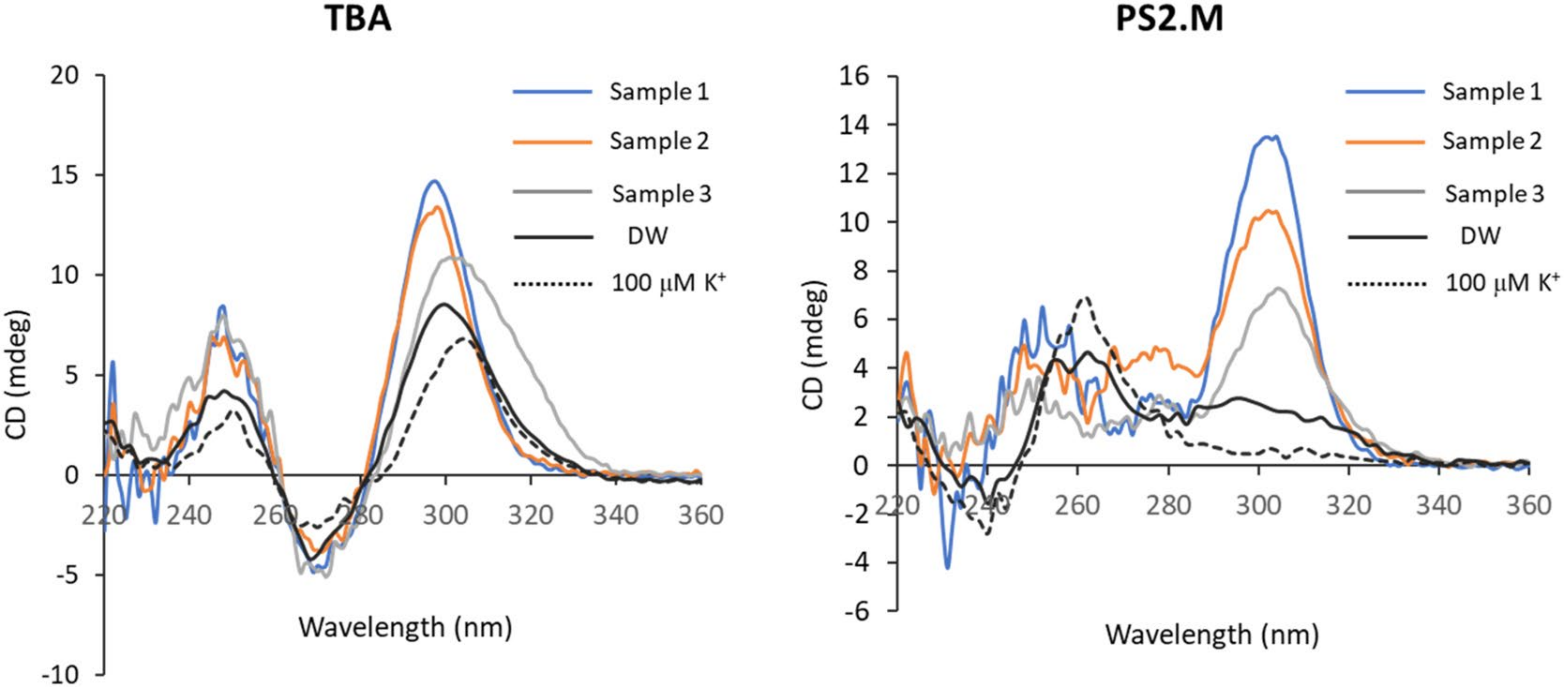
CD spectra of TBA and PS2.M added by three effluent samples (Sample 1–3), DW and 100 μM K^+^.

Figure 9 shows that the CD spectra of TBA and PS2.M added in effluent samples. The concentration of Pb^2+^ in Sample 1 and Sample 2 was 3.30 ppb and 1.40 ppb, respectively, but not detected in Sample 3. In case of PS2.M, the three effluent samples without spiked Pb^2+^ caused a discrete shift of the absorption band to the Pb^2+^-specific wavelength, 314 nm. This result indicated that the Pb^2+^ in the effluent sample as low as 3.3 ppb (0.0159 μM) could convert PS2.M to a G-QD structure, and be simply measured by CD spectroscopy without any other platform and labeling reagents. Therefore, PS2.M could be more promising as a detection probes for Pb^2+^ in environmental samples, because they caused a suitable absorption signal in CD spectroscopy.

The limits of permissible concentration of Pb^2+^ in surface water are as follows: 50 ppb for drinking water supply^41^; 15 ppb for drinking water^42^; 50–100 ppb for permissible effluent water^43^; and 200–500 ppm industrial wastewater^36^. In Fig. 9, the CD intensity at 314 nm by PS2.M could be related to the concentration of Pb^2+^, measured by ICP-OES. Furthermore, the PS2.M was applicable to detect the level of Pb^2+^ concentration permissible for drinking water supply (50 ppb) and for drinking water (15 ppb).

Comparing to other methods to determine the low concentration of Pb^2+^, ICP-OES and Inductively Coupled Plasma Mass Spectrometer (ICP-MS) could be applied. The detection limit of Pb^2+^ by ICP-OES and ICP-MS is known nearly as 5 ppb and 5 ppt, respectively, but the required volume of samples is 50–100 mL for each instrument. In addition, the operation of both instruments is difficult compared to CD spectroscopy. One of the G-QDs studied in this study, PS2.M, could detect the Pb^2+^ in environmental samples below 5 ppb using 2 mL volume.

Along with K^+^, Na^+^ could be also contaminated in environmental samples, which is known to be intercalated into the nucleotides to convert to G-QDs. However, PS2.M was reported to show no response to Na^+^^24^, and TBA could be converted to G-QDs by Na^+^ but the spectra converted by Na^+^ was similar to that of K^+^^44^.

## Conclusion

To determine the performance of G-QDs as a probe for Pb^2+^ detection, five types of nucleotides were compared for their binding affinity. Two of them, TBA and PS2.M, which showed higher binding constants, were analyzed for their specificity and applicability for environmental sensors to detect Pb^2+^. Although K^+^ has been reported to have a strong binding affinity to G-QDs, we found that the coexistence of Pb^2+^ with K^+^ concentrations up to 100 μM has a negligible effect on Pb^2+^ binding. The binding constants of TBA and PS2.M were comparable to those of the antigen–antibody reaction, and the Pb^2+^ detection property of PS2.M was suitable for the analysis of environmental water.

In this study, several G-QDs were thoroughly examined for the binding constant for Pb^2+^ and cross-reactivity to other metal ions, which were crucial data for the application of them to platform- and labeling-free detection of Pb^2+^. The results demonstrated the substantial role of the structural transition of TBA and PS2.M in sensing the Pb^2+^ concentration. Furthermore, in a complex sample such as an effluent discharged from WWTP, solubilized Pb^2+^ was detected below 5 ppb levels. Therefore, the proposed method has promising applications in environmental and laboratory sample analysis, because of the simple and cost-effective preparation of G-QDs and detection procedures.

## Supplementary information

Supplementary Information.

## References

[1] Tchounwou PB, Yedjou CG, Patlolla AK, Sutton DJ (2012). Heavy metal toxicity and the environment. Exp. Suppl..

[2] Tam NFY, Wong YS, Wong MH (1988). Heavy metal contamination by Al-fabrication plants in Hong Kong. Environ. Int..

[3] Vouk VB, Piver WT (1983). Metallic elements in fossil fuel combustion products: Amounts and form of emissions and evaluation of carcinogenicity and mutagenicity. Environ. Health Perspect..

[4] Al-Musharafi SK (2016). Heavy metals in sewage treated effluents: Pollution and microbial bioremediation from arid regions. Open Biotechnol. J..

[5] Su P (2020). Pb-based perovskite solar cells and the underlying pollution behind clean energy: Dynamic leaching of toxic substances from discarded perovskite solar cells. J. Phys. Chem. Lett..

[6] Hildebrandt, T., Osada, A., Peng, S. & Moyer, T. J. Standards and tests for lead–acid batteries in automotive applications In *Lead-Acid Batteries for Future Automobiles* (eds. Garche, J., Karden, E., Moseley, P. T. & Rand, D. A. J.) 551–573 (Elsevier, Amsterdam, 2017).

[7] Deb P, Jamison R, Mong L, Paul U (2015). An evaluation of the shielding effectiveness of lead aprons used in clinics for protection against ionising radiation from novel radioisotopes. Radiat. Prot. Dosimetry.

[8] Alya A, Ines DB, Montassar L, Najoua G, Saloua EF (2015). Oxidative stress, biochemical alterations, and hyperlipidemia in female rats induced by lead chronic toxicity during puberty and post puberty periods. Iran J. Basic Med. Sci..

[9] Begovic V, Nozic D, Kupresanin S, Tarabar D (2008). Extreme gastric dilation caused by chronic lead poisoning: A case report. World J. Gastroenterol..

[10] Wani AL, Ara A, Usmani JA (2015). Lead toxicity: A review. Interdiscip. Toxicol..

[11] Nakata H (2017). Monitoring lead (Pb) pollution and identifying Pb pollution sources in Japan using stable Pb isotope analysis with kidneys of wild rats. Int. J. Environ. Res. Public Health.

[12] Bochman ML, Paeschke K, Zakian VA (2012). DNA secondary structures: Stability and function of G-quadruplex structures. Nat. Rev. Genet..

[13] Tian T, Chen YQ, Wang SR, Zhou X (2018). G-Quadruplex: A regulator of gene expression and its chemical targeting. Chem..

[14] Bhattacharyya D, Mirihana Arachchilage G, Basu S (2016). Metal cations in G-quadruplex folding and stability. Front. Chem..

[15] Ida J (2019). G-quadruplexes as an alternative recognition element in disease-related target sensing. Molecules.

[16] Tan J, Lan L (2020). The DNA secondary structures at telomeres and genome instability. Cell Biosci..

[17] Dolot R (2018). Crystal structures of thrombin in complex with chemically modified thrombin DNA aptamers reveal the origins of enhanced affinity. Nucleic Acids Res..

[18] Bruni L, Manghi M, Croci S (2018). PS2.M: Looking for a potassium biosensor. Eur. Phys. J. Plus.

[19] Lim KW (2009). Structure of the human telomere in K^+^ solution: A stable basket-type G-quadruplex with only two G-tetrad layers. J. Am. Chem. Soc..

[20] Do NQ, Chung WJ, Truong THA, Heddi B, Phan AT (2017). G-quadruplex structure of an anti-proliferative DNA sequence. Nucleic Acids Res..

[21] Liu W (2012). G-quadruplex formation and sequence effect on the assembly of G-rich oligonucleotides induced by Pb^2+^ ions. Soft Matter.

[22] Yu Z (2018). Insights into the competition between K^+^ and Pb^2+^ binding to a G-quadruplex and discovery of a novel K^+^–Pb^2+^–quadruplex intermediate. J. Phys. Chem. B.

[23] Liu W (2011). Kinetics and mechanism of conformational changes in a G-quadruplex of thrombin-binding aptamer induced by Pb^2+^. J. Phys. Chem. B.

[24] Liu W (2012). Kinetics and mechanism of G-quadruplex formation and conformational switch in a G-quadruplex of PS2.M induced by Pb2+. Nucleic Acids Res..

[25] Li S (2018). Detection of radon with biosensors based on the lead(II)-induced conformational change of aptamer HTG and malachite green fluorescence probe. J. Environ. Radioact..

[26] Liang G (2017). DNAzyme-based biosensor for detection of lead ion: A review. Microchem. J..

[27] Peng D (2019). Efficient DNA-catalyzed porphyrin metalation for fluorescent ratiometric Pb^2+^ detection. Anal. Chem..

[28] Wang Z (2016). Phosphate-perylene modified G-quadruplex probes for the detection of Pb2+ using fluorescence anisotropy. J. Mater. Chem. B..

[29] Ye T (2019). Polarity inversion sensitized G-quadruplex metal sensors with K+ tolerance. Biosens. Bioelectron..

[30] Kypr J, Kejnovská I, Renčiuk D, Vorlíčková M (2009). Circular dichroism and conformational polymorphism of DNA. Nucleic Acids Res..

[31] Manna S, Srivatsan SG (2018). Fluorescence-based tools to probe G-quadruplexes in cell-free and cellular environments. RSC Adv..

[32] Umar MI, Ji D, Chan C-Y, Kwok CK (2019). G-quadruplex-based fluorescent turn-on ligands and aptamers: From development to applications. Molecules.

[33] Tseng T-Y (2018). The G-quadruplex fluorescent probe 3,6-bis(1-methyl-2-vinyl-pyridinium) carbazole diiodide as a biosensor for human cancers. Sci. Rep..

[34] Pagano B, Martino L, Randazzo A, Giancola C (2008). Stability and binding properties of a modified thrombin binding aptamer. Biophys. J..

[35] Bianchi F (2018). Structure of human telomere G-quadruplex in the presence of a model drug along the thermal unfolding pathway. Nucleic Acids Res..

[36] Zhang L-M, Cui Y-M, Zhu L-N, Chu J-Q, Kong D-M (2019). Cationic porphyrins with large side arm substituents as resonance light scattering ratiometric probes for specific recognition of nucleic acid G-quadruplexes. Nucleic Acids Res..

[37] Demkovičová E (2017). Telomeric G-quadruplexes: From human to tetrahymena repeats. J. Nucleic Acids..

[38] Czikkely M, Neubauer E, Fekete I, Ymeri P, Fogarassy C (2018). Review of heavy metal adsorption processes by several organic matters from wastewaters. Water.

[39] Kamaruddin NH, Bakar AAA, Mobarak NN, Zan MSD, Arsad N (2017). Binding affinity of a highly sensitive Au/Ag/Au/chitosan-graphene oxide sensor based on direct detection of Pb^2+^ and Hg^2+^ ions. Sensors (Basel).

[40] Singh R, Das G (2019). “Turn-on” Pb^2+^ sensing and rapid detection of biothiols in aqueous medium and real samples. Analyst.

[41] OECD. *The Environmental Action Program Task Force Secretariat for UK DEFRA.* https://www.oecd.org/env/outreach/38205453.pdf (2007).

[42] Dignam T, Kaufmann RB, LeStourgeon L, Brown MJ (2019). Control of lead sources in the United States, 1970–2017: Public health progress and current challenges to eliminating lead exposure. J. Public Health Manag. Pract..

[43] Arbabi M, Hemati S, Amiri M (2015). Removal of lead ions from industrial wastewater: A review of Removal methods. Int. J. Epidemiol. Res..

[44] Świtalska A (2018). Cholesterol-bearing fluorescent G-quadruplex potassium probes for anchoring at the langmuir monolayer and cell membrane. Sensor (Basel).

