# Supplementary material for: Platform- and label-free detection of lead ions in environmental and laboratory samples using G-quadraplex probes by circular dichroism spectroscopy
Source: Sci Rep. 2020 Nov 24;10:20461. doi: 10.1038/s41598-020-77449-5 (PMC7686487; doi:10.1038/s41598-020-77449-5)
Supplement: Supplementary file 1 — Supplementary Information. [file 41598_2020_77449_MOESM1_ESM.docx]

*Supplementary Information*

**Platform- and label-free detection of lead ions in environmental and laboratory samples using G-quadraplex probes by circular dichroism spectroscopy**

Raeyeong Kim,^1^ Young-SangYoun,^1^ Misook Kang,^1*^ and Eunjoo Kim^2*^

^1^Department of Chemistry, Yeungnam University, Gyeonsan-city, Gyeongbuk 38541, Republic of Korea.

^2^Division of Electronic Information System, Daegu Gyeongbuk Institute of Science and Technology, Techno-jungangdaero 333, Daegu, Republic of Korea, 42988

^*^Correspondence to :

Eunjoo Kim ([ejkim@dgist.ac.kr](mailto:ejkim@dgist.ac.kr))

Misook Kang([mskang@ynu.ac.kr](mailto:mskang@ynu.ac.kr))


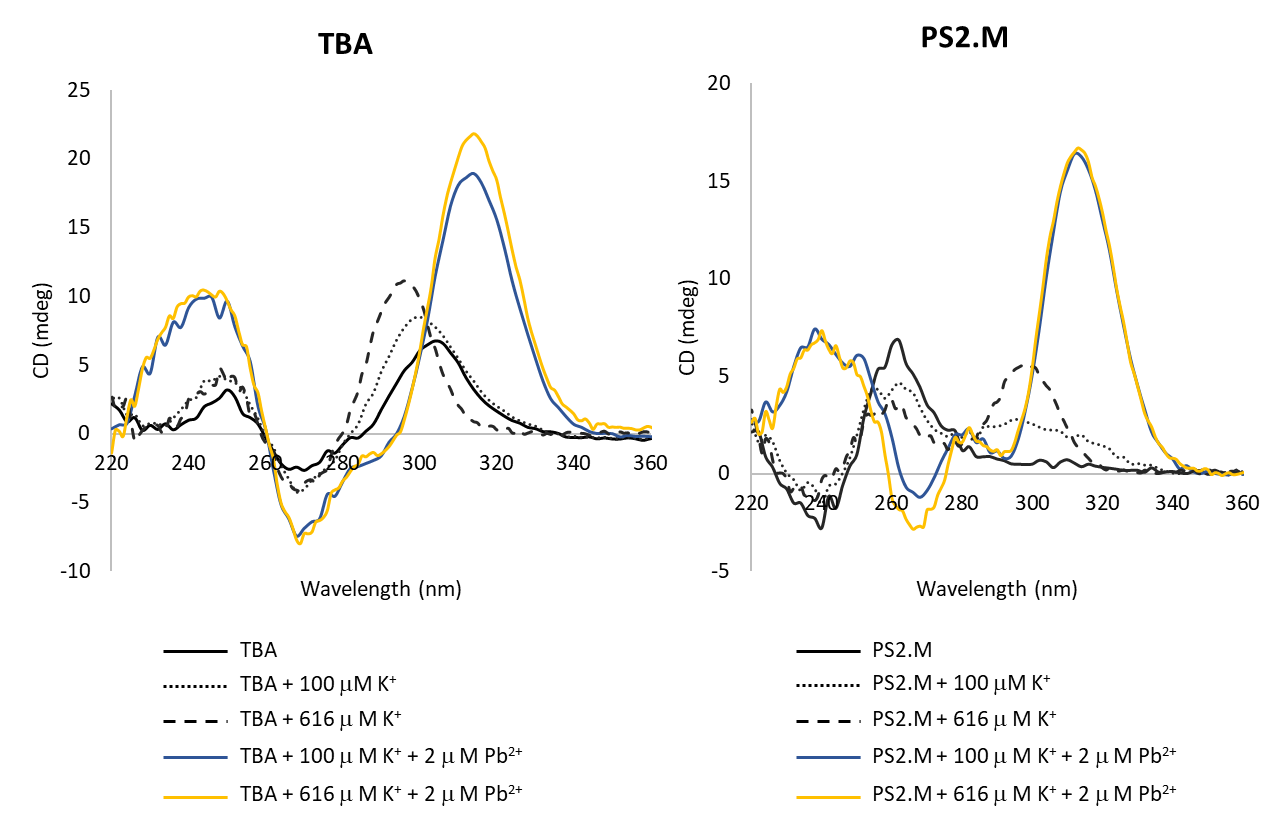


Figure S1. CD spectra of TBA and PS2.M by 100 μM and 616 μM K^+^. The K^+^ concentration in Sample 1 effluent was 616 μM, and the spectral intensity was comparable to that of 100 μM K^+^.
